# Effectiveness and Preparedness of Institutions' E-Learning Methods During the COVID-19 Pandemic for Residents' Medical Training in Saudi Arabia: A Pilot Study

**DOI:** 10.3389/fpubh.2021.707833

**Published:** 2021-08-30

**Authors:** Basim Alsaywid, Miltiadis D. Lytras, Maha Abuzenada, Hara Lytra, Lama Sultan, Hala Badawoud, Wesam Abuznadah, Sami A. Alhaider, Abdulrahman Housawi, Areti Apostolaki

**Affiliations:** ^1^Planning and Organizational Excellence Administration, Saudi Commission for Health Specialties, Riyadh, Saudi Arabia; ^2^King Abdulaziz Medical City, Ministry of National Guard, Jeddah, Saudi Arabia; ^3^College of Medicine, King Saud Bin Abdulaziz University for Health Sciences, Ministry of National Guard, Jeddah, Saudi Arabia; ^4^Effat College of Engineering, Effat University, Jeddah, Saudi Arabia; ^5^Distinguished Scientists Program, Faculty of Computing and Information Technology, King Abdulaziz UniversityJeddah, Saudi Arabia; ^6^School of Medicine, University of Patras, Patras, Greece; ^7^Saudi Commission for Health Specialties, Riyadh, Saudi Arabia; ^8^Health and Welfare Units Administration, Technological, Educational Institute of Peloponnese, Kalamata, Greece

**Keywords:** e-learning, COVID-19, medical training, effectiveness and efficiency, public health, Saudi Commission for Health Specialties (SCFHS)

## Abstract

**Background:** Under the urgent circumstances of the COVID-19 pandemic, higher education institutions of an international scale have resorted to online education methods, exclusive or not. Among those, medical institutions are under double pressure, fighting the pandemic's effects and, at the same time providing efficient clinical training to their residents. The main aim of the study is to evaluate the preparedness of the educational institutions for the e-learning platform transition for the delivery of medical training and also to evaluate the overall satisfaction level of the participants with their e-learning experience.

**Methods:** This is an observational cross-sectional study design. The survey's sample included 300 medical students and residents of multiple training levels and specialties, coming from more than 15 different cities of Saudi Arabia. Filling the questionnaire required specific inclusion criteria and all obtained data were secured by the Saudi Commission of Health specialty. The main objective was to evaluate the quality of e-learning methods provided by medical universities. For the collection of the data, Survey Monkey software was used and the analysis was conducted with SPSS.

**Results:** The study found that the frequency of digital education use increased by ~61% during the coronavirus crisis, while almost 9 out of 10 residents have used some e-learning platform. It was reported that before the pandemic, participants' online training was deemed to be rather ineffective, given the rate of 3.65 out of 10. However, despite the increase in e-learning use after COVID-19, many obstacles arose duringcthe adaptation process. According to our survey: lectures and training sessions were not conducted as per the curriculum (56.33%); both students and instructors' academic behavior and attitude changed (48.33%); engagement, satisfaction, and motivation in class were rated low (5.93, 6.33, and 6.54 out of 10 accordingly), compared to the desired ones. Still, participants accredited e-learning as a potential mandatory tool (77.67%) and pinpointed the qualifications that in their opinion will maximize educational impact.

**Conclusion:** The study concluded that innovative restructuring of online education should be based on defined critical success factors (technical support, content enhancement, pedagogy etc.) and if possible, set priority levels, so that a more permanent e-learning practice is achievable. Also our study confirmed that students were overall satisfied with the e-learning support of the training method.

## Introduction

The global spread of the novel coronavirus (COVID-19) has emerged in late December 2019 keeping billions locked down at home. The Covid-19 disease has been labeled by the World Health Organization (WHO) as an international public health emergency (1). The Saudi government and the public health experts took very tight measures to minimize the risk of uncontrollable spread of the infection. In response to this pandemic, several countries have announced the closure of all educational institutions. To ensure that students finish their curriculum on time, universities have moved rapidly to transition various courses and programs from on-site to an online delivery model. Education has changed dramatically, with the distinctive rise of e-learning and on digital platforms (2).

Globally, online learning is not a new method of teaching for any university but preparedness for the pandemic crisis varies between institutions. Many faculty members have been trained to use online learning platforms as an add-on to on-site teaching. Online working at home may be a difficult task due to the high demand of using computers from the whole family (3). These rapid transformations have raised questions about faculties' capability to deal with their existing technology and resources, but they have also provoked surprising new models of educational innovation (3).

However, medical institutions are also under pressures at this uncertain period and there are a lot of challenges to deal with in training healthcare professions with the limitation of social distancing. Some clinical training, simulations, technology sessions, and laboratory sessions cannot be delivered remotely and not all learners have internet access or computers at home (4). Examinations have also transitioned to online settings. For residents and fellows, canceling elective and non-urgent surgeries and routine appointments, reducing inpatients admission, and shortage of personal protective equipment (PPE) my negatively impact the quality of their clinical practice (5).

In the Saudi Commission for the Health Specialties (SCFHS), we recently delivered an integrated research related to the evaluation of Residents' Training in COVID-19 Pandemic Times with an emphasis on Educational Process, Institutional Support, Anxiety, and Depression (6). The assessment of e-learning delivery in this study can be also exploited in the direction of informing our recently published set of Key Performance Indicators (KPIs) for Sustainable Post-graduate Medical Training: toward the Implementation of an Innovative Approach to Advance the Quality of Training Programs at the Saudi Commission for Health Specialties (SCFHS) (7). Furthermore, the analysis of quality benchmarks and the interpretations of e-learning perceptions of students in residency post-graduate medical training programs is a key priority (8), with an emphasis on change in management (9) and sophisticated e-learning strategies capable of dealing with the need for personalized learning services (10).

In a recent study Yang et al. (11), provided an integrated research paper on the preparedness of medical education in China with an emphasis on the lessons learnt from the COVID-19 outbreak. Their work reaveled three significant factors that need to be addressed from an educational point of view: insufficient emphasis on public health emergency preparedness; unsophisticated mechanisms for interdisciplinary cooperation; and inadequate guidance in medical ethics.

Within the challenging context of COVID-19 pandemic, educational institutions also had to adjust their learning strategies, and to adapt the educational practice to the new reality.

Shehata et al. (12), in their recent work, focus on the required educational adjustment in the context of medical training in Egypt. The focus of their analysis and their discussion covered a variety of medical education aspects including, among others, the University preparedness, the role of faculty in the transition, as well as the Role of ME units/Departments/National/Regional bodies in the transition. One of the key findings of their work was the supportive opinions of participants for the integration of online learning activities to the future curriculum in post-COVID-19 times.

Along with the emphasis on the preparedness of institutions, much research focuses on aspects related to medical students' preparedness for the COVID-19 pandemic (13). O'Byrne et al. analyzed the medical students' mental health and concluded that there is a critical need for “pandemic preparedness” to be embedded in the medical curriculum. Other studies in the literature also emphasize residency post-graduate programs (14). Chong et al., in this context, contributed to a thorough analysis of the leadership requirements for radiology residency programs in order to manage effectively the residency-related impact of the pandemic with special emphasis to be placed on education. In the same context, the interesting work of Choi et al. (15), analyzes how the COVID-19 pandemic challenges the final year medical students in the United Kingdom with emphasis on final examinations and placements. Their study, including 440 students from 32 UK medical schools, proved that 38.4% (*n* = 169) of respondents had their final OSCEs canceled while 43.0% (*n* = 189) had already completed their final OSCEs before restrictions. 43.0% (*n* = 189) of assistantship placements were post-poned while 77.3% (*n* = 340) had electives canceled. The main contribution of the study is to reveal the key aspects of the disruptions to students' support with a key effect on the students' preparedness. The study also concludes by highlighting the crucial role of the well-being of students in their mental health status.

Mukhtar et al. (16) contributed to the relevant discussion by analyzing the advantages, limitations, and recommendation for e-learning during COVID-19 with a focus on Pakistan. The main advantages of the transition and the delivery of training online were clarified to be the comfort and the accessibility. One of the key limitations was proved to be the barriers in maintaining academic integrity. In their study, they also discuss the key need to reduce the cognitive load of online content and to increase the interactivity.

The key objective of sustainability is also well-discussed in the relevant literature. For example, in their study, Ashokka et al. (17) are promoting the discussion on sustaining medical education during COVID-19 with an emphasis on responses of medical training centers.

The issue of engagement and active learning during COVID-19 has also been studied by various researchers (18). In this study, the authors provide concrete ideas and lessons learnt related to the enhancement of the students' engagement in the context of online delivery of education. Almarzooq et al. (19) also consider e-learning as a disruptive technology and methodology for the graduate medical education. They also make reference to the American College of Cardiology Fellowsin-Training Section Leadership Council which proposed three novel educational strategies: personalized learning, adaptive learning with real-world situations and feedback, and the flipped classroom. Their final comment is that during the pandemic the acceleration of these strategies is challenging the efficiency and the quality of medical training.

Sahi et al. (20) also comment on the need during the COVID-19 pandemic for continuity of medical education with emphasis on pedagogical innovations involving technology and simulation-based teaching. Arandjelovic et al. (21), in a thorough study, review the strategies implemented during previous global infectious disease epidemics and suggest strategies for utilizing clinical education in times of pandemic crisis. Similar discussion and arguments for the support of medical education with e-learning can be found in (22, 23) with emphasis in multimodal multi-Institutional solution to remote medical student education and Digitalization or strategic consideration of key issues like the Strategic Deployment of Cardiology Fellows in Training Using the Accreditation Council for Graduate Medical Education Coronavirus Disease (24).

In Table 1, below, we provided the key aspects of the studies found in the literature and in Table 2, we summarize an overview of the main aspects of focus.

**Table 1:** An overview of our critical literature review and key aspects of the research problem.

	**Literature review**		
**References**	**Title of article**	**Key contribution**	**Impact on our research model/connection to our research aims**
Yang et al. (11)	Preparedness of medical education in China: Lessons from the COVID-19 outbreak.	On the one hand, we believe that the distinguished contributions in disease containment efforts by healthcare professionals indicated that our medical education system has achieved its intended outcomes and is socially accountable. On the other hand, we have also identified three major issues that need to be addressed from an educational standpoint: insufficient emphasis on public health emergency preparedness; unsophisticated mechanisms for interdisciplinary cooperation; and inadequate guidance in medical ethics.	•Focus on preparedness •Lessons Learnt
Shehata et al. (12)	Medical education adaptations post-COVID-19: an Egyptian reflection.	The study aims to explore how medical schools in Egypt responded to COVID-19 pandemic regarding teaching and learning/assessment for undergraduate students.	•Adjustments to medical training •Provision of Assessments
O'Byrne et al. (13)	Medical students and COVID-19: the need for pandemic preparedness.	With the available evidence suggesting that medical students' mental health status is already poorer than that of the general population, with academic stress being a chief predictor, such changes are likely to have a significant effect on these students. This, in conjunction with the likelihood of future pandemics, highlights the need for ‘pandemic preparedness' to be embedded in the medical curriculum.	•Students' mental health •Integration of pandemic preparedness to Curriculum
Chong et al. (14)	Radiology residency preparedness and response to the COVID-19 pandemic.	The aim of this article is to provide specific guidance for radiology residency program leadership to prepare and respond to the residency-related impact from the pandemic, with focus on safety and education.	•Impact of pandemic to residency programs
Choi et al. (15)	The impact of the COVID-19 pandemic on final year medical students in the United Kingdom: a national survey.	The aim of this study is to identify the impact of COVID-19 on final year medical students' examinations and placements in the United Kingdom (UK) and how it might impact their confidence and preparedness going into their first year of foundation training.	•Final year medical students examinations and placements.
Mukhtar et al. (16)	Advantages, Limitations and Recommendations for online learning during COVID-19 pandemic era.	The current study supports the use of online learning in medical and dental institutes, considering its various advantages.	•E-learning best practices •E-learning strategies
Ashokka et al. (17)	Coordinated responses of academic medical centers to pandemics: sustaining medical education during COVID-19.	Major themes of medical education management include leveraging on remote or decentralized modes of medical education delivery, maintaining the integrity of formative and summative assessments while restructuring patient-contact components, and developing action plans for maintenance of essential activities based on pandemic risk alert levels.	•Sustaining Education •Sustainability of medical training in times of pandemic
Zayapragassarazan et al. (18)	COVID-19: Strategies for Engaging Remote Learners in Medical Education.	This research discusses about the different strategies for ensuring higher levels of online student engagement in medical education.	•Investigation of active learning strategies for engagement of learners In COVID-19 times
Almarzooq et al. (19)	Virtual learning during the COVID-19 pandemic: a disruptive technology in graduate medical education.	In this paper, we describe the capabilities, implementation, and challenges of virtual learning for cardiology fellows-intraining (FITs) and fellowship programs in the COVID-19 era and beyond.	•Understanding the disruptive character of e-learning platform for medical training during COVID-19
Sahi et al. (20)	Medical education amid the COVID-19 pandemic, Indian pediatrics.	In this pandemic, the need for uninterrupted generation of future doctors is felt more than ever in our living memory. Continuity of medical education is thus imperative. While “Live” patient contact is an irreplaceable tenet of clinical teaching, these extraordinary times demand exceptional measures. Pedagogical innovations involving technology and simulation based teaching (Online lectures, video case vignettes, virtual simulators, webcasting, online chat-rooms) need to be brought to the forefront.	Understanding the kind of educational innovations required in pandemic times for medical training •Testing the efficiency of e-learning method to deliver the learning objectives and to secure the quality of education and training
Arandjelovic et al. (21)	COVID-19: considerations for medical education during a pandemic.	We discuss the considerations behind these changes, review the strategies implemented during previous global infectious disease epidemics, and suggest strategies for maximizing clinical education going forward.	Justifying the e-learning strategy and the components required for an effective e-learning support for medical training in times of COVID-19 pandemic
Ruthberg et al. (22)	Solution to Remote Medical Student Education for Otolaryngology during Covid-19.	In this commentary, we discuss the multi-institutional development of a robust syllabus for medical students using a multimodal collection of resources. Medical students collaborated with faculty and residents from 2 major academic centers to identify essential otolaryngology topics.	Understudying the required enhancement and revisions in curriculum to address COVID-19 pandemic challenges.
Alkhowailed et al. (23)	Digitalization Plan in Medical Education during Covid-19 Lockdown.	The present descriptive cross-sectional study was conducted to reveal the different digital procedures implemented by the College of Medicine at Qassim University for better student performance and achievement.	Analyzing the multi-faced revisions of procedures and policies to enable students' efficiency and performance during COVID-19 pandemic
Gallagher et al. (24)	Strategic Deployment of Cardiology Fellows in Training Using the Accreditation Council for Graduate Medical Education Coronavirus Disease 2019 Framework.	The purpose of this review is to describe our departmental strategic deployment of cardiology fellows in training using the Accreditation Council for Graduate Medical Education framework for pandemic preparedness.	Linking e-learning method to integrated performance framework

**Table 2:** Key focus of selected literature.

	**Key emphasis**					
**References**	**Covid-19**	**Medical education**	**Public health emergency**	**Preparedness**	**Medical ethics**	**Online learning**
Yang et al. (11)	x	x	x	x	x	
Shehata et al. (12)	x	x		XNew teaching and assessment methods		x
O'Byrne et al. (13)	x	x	X Volunteering	x	X Moral trauma	
Chong et al. (14)	x	XRadiology education		x	X Anxiety and Well-being Issues of Trainees	
Choi et al. (5)	x	x		x		x
Mukhtar et al. (16)	x	XUndergraduate				x
Ashokka et al. (17)		XUndergraduate	x			Xe-learning/computer
Zayapragassaraza (18)	x	x			x	x
Almarzooq et al. (19)	x	x		XIntegration		XCommunicationCollaboration
Sahi et al. (20)	x	x		XPedagogical innovations		x
Arandjelovic et al. (21)	x	x	X Medical students on the frontline	XChanges to medical schools during a pandemic		
Ruthberg et al. (22)	x	XOtolarynologyActing internship		XMultimodal		XRemote education
Alkhowailed et al. (23)	x	x		XDigitalization		XVirtual classroom
Gallagher et al. (24)	x	XFellows in training	X Health services			

## Methods

In this research study we adopted an integrated research method aiming to promote the following four primary research objectives:

- To evaluate the preparedness of the educational institutions for the e-learning platform transition for the delivery of medical training
- To evaluate the effectiveness of the e-learning educational method among undergraduate and post-graduate education of health professions
- To evaluate the overall satisfaction level of the participants with their e-learning experience
- To review methods of assessment in the e-learning platform and to understand their suitability and effectiveness.

The research setting was targeted to all training hospitals and universities across in the country. In this research paper we focus on a pilot study among 300 participants. The inclusion criteria for the participants are related to undergraduate students and post-graduate trainees from all health disciplines (medicine, dentistry, applied health sciences, pharmacy, and nursing) from both genders in all levels of training. The exclusion criteria refer to all residents who are on leave or outside rotation. The research design is related to an observational cross sectional study design. Participants who fulfill the inclusion criteria were invited to participate in this study by filling in an online self-administered closed ended questionnaire about the effectiveness of the online educational method and the preparedness of the institutions for such an activity.

The questionnaire has the following sections:

- Section 1: Addressing the demographic data of the participants which include Age, City, Institution, College, Job title and Subspecialty.
- Section 2: E-learning experience before the COVID-19 pandemic
- Section 3: E-learning experience during the COVID-19 pandemic
- Section 4: Review methods of assessment in the e-learning platform

In the section Results we provide a statistical analysis summarized and analyzed using SPSS program. Simple descriptive statistics are reported in the form of mean and standard deviation for frequency, and percentage for qualitative data.

We also deployed qualitative analysis and sentiment analysis for analyzing the open text contributions of participants related to their perceptions for the adoption of e-learning in medical training. For the Ethical consideration of our study the main rule is that participants' confidentiality will be kept and they will not be forced to fill out the questionnaire. Name and contact numbers were not requested. All data are secured in the Saudi Commission of Health Specialties' computer facility. Data in the next section are summarized and presented with honesty and without any falsification or fabrication.

In our research a total number of 300 respondents participated, meeting the inclusion criteria presented in the previous section. In total, 122 of them (41%) were female and 178 (59%) male. Concerning the training level of participants as indicated in Table 3, residents of R1, R2, and R3 level, are representing almost 65% of the respondents. The basic demographic data of our study are summarized in Tables 3, 4 below.

**Table 3:** Training level of participants.

**Training Level**	**Count**	**Percentage (%)**
F1	14	4.7
F2	7	2.3
F3	2	0.7
R1	84	28.0
R2	51	17.0
R3	54	18.0
R4	37	12.3
R5	13	4.3
I am a RTP	36	12.0
Not available	2	0.7
Total	300	100.0

**Table 4:** Training city of participants.

**Training city**	**Count**	**Percentage (%)**
Abha	15	5.0
Al-baha	3	1.0
Al-hasa	21	7.0
Al-jouf	1	0.3
Al-khobar	25	8.3
Al-Madina Al-Monawara	10	3.3
Al-quaseem	4	1.3
Dahran	5	1.7
Dammam	27	9.0
Jezan	12	4.0
Jubail	3	1.0
Makkah Al-Mokarrma	83	27.7
Najran	4	1.3
Qatif	9	3.0
Riyadh	63	21.0
Tabouk	3	1.0
Taif	7	2.3
Yanbu	3	1.0
Not available	2	0.7
	300	100.0

As per the training city in Table 4, there is an overview of the respondent's location. Makkah Al-Mokarrma and Riyadh is the basis for almost 49% of the participants in our survey. In the next section we provide the empirical data analysis of our research study. In this paper we present the key facts and interpretation of descriptive statistics. In another research we will elaborate with advanced statistical analysis and multi-variate analysis. The main effort in this research is to document and discuss the qualitative aspects of the experiences of trainees in COVID-19 times and to understand the lessons learnt.

## Results

### Perceptions and Attitudes of Participants for E-Learning Experience

One of the key objectives of our research is to understand and to interpret the effectiveness and the readiness of the medical training centers of residents to adopt e-learning platforms and online instruction during the COVID-19. In Table 5, below, we provide the key findings. Participants in our survey were asked to rate their attitude and perception in a scale of 1 to 10, where one represented the lowest perception or use and 10 the highest perception or use.

**Table 5:** Perceptions and attitudes of participants for e-learning experience.

**Question number**	**Rating (1–10)**
Q6. Frequency of E-learning at institution before COVID-19	3.55
Q7. How Efficient your institutional uses of e-learning platform before COVID-19	3.65
Q10. On-site education experience during COVID-19	5.50
Q11. Rating off-site education experience during COVID-19	6.05
Q14. Frequency of E-learning at institution during COVID-19	5.71

The residents' training medical centers were used at a rather low rate for e-learning methods before the COVID-19 pandemic. This reality was enhanced significantly during the COVID-19 pandemic, and the frequency of e-learning use at institutions was increased at a bold rate, from 3.55 to 5.71 (Q6), as seen in Table 5, above (increase by ~61%).

Another significant finding is that the participants in our survey rate rather low the efficiency of the institutional use of e-learning platform before COVID-19. The average rating of 3.65 out of 10 indicates that most of the participants were not satisfied with the use of the e-learning platform in their institutions before the COVID-19 pandemic (Q7).

As per the evaluation of respondents for the on-site and the off-site education experience during COVID-19, the key findings permit interpretation. The off-site education experience during COVID-19 was rated 10% higher than the on-site education experience (6.05/10 compared to 5.50 respectively; Q11 and Q10). We will discuss the limitation of our study in the next section, but a first comment and interpretation of this perception is that trainees prefer the off-site training to the on-site. For sure, this simplistic assumption requires further analysis. We do have to understand which components of the e-learning educational approach are valued by trainees and our respondents.

The main facts related to the transition of instruction during COVID-19 are presented in Table 6, below.

**Table 6:** Adjustment to e-learning environment during the pandemic.

**Question Number**	**Yes (%)**	**No (%)**
Q12. Have you used e-learning platform during COVID-19 era?	89.33	10.67
Q13. Since COVID-19 pandemic, do your education activities still conducted as per curriculum?	56.33	43.67
Q23. Have you observed any changes in student's/faculty behavior or personality during e-learning experience?	48.33	51.67
Q24. Do you think e-learning should be implemented as a mandatory educational tool in future?	77.67	22.33

The need of medical training institutions in Saudi Arabia to adapt quickly to the new reality of COVID-19 resulted in the extensive use of e-learning platforms. Almost 9 out of 10 respondents acknowledged the use of the e-learning platform during the pandemic (Q12).

The disruption in educational activities per curriculum was also significant. According to 44% of our respondents, the educational activities stopped being conducted as per the curriculum (Q13). This fact requires further investigation concerning the real impact of the e-learning mode on the quality of education and the skills and competencies of the residents.

Additionally, it is extremely important to understand the impact of the e-learning delivery on the behavior and personality of students and faculty. The psychological pressure and requirement for the adoption of digital and other soft skills, related to online delivery of education, need to be analyzed further. Our survey participants confirmed at a very high rate (almost 50%) that they observed some changes in students' or faculty's behavior during e-learning experience (Q23).

Our respondents also answered that e-learning practice should be an inevitable or useful pillar of any future training approach. Despite the difficulties and barriers of the on-site e-learning mode, trainees realized the additional value of the online approach; not as an exclusive mode of medical training but as a supporting, enhancing enabler. Thus, in the relevant question on their opinion of whether e-learning should be implemented as a mandatory educational tool in the future, 77.67% of them foresee its future potential (Q24).

### Attitude of Participants for the Value Adding Components of the E-Learning Mode During COVID-19 for Medical Training

In Table 7 below, we present the results related to the attitude and perceptions of trainees for the value-adding components of the e-learning mode. It is evident that the trainees during COVID-19 confirm a rather moderate engagement in lectures through e-learning platforms. Their evaluation on this factor is rated with 5.93 out of 10. This key finding can initiate educational strategies that can be facilitated by digital means and can promote the active participation of students. Various propositions for active learning and medical training can be found in the relevant literature. Also, it is extremely important to analyze and to provide creative ideas for the simulation of clinical and surgical training and practice in times of pandemics like COVID-19. This period is somehow a test bed for best practices, lessons learnt, and creative policy making for the future.

**Table 7:** Attitude of participants for the value adding components of the E-learning mode during COVID-19 for medical training.

**Question number**	**Rating (1–10)**
Q17. Rating of engagement during Covid-19	5.93
Q18. Rating of overall satisfaction related to the use of e-learning platform during COVID-19	6.33
Q19. Rating of your motivation to start -e-learning during COVID-19	6.54
Q20. Rating of barriers faced during E-learning mode of instruction during COVID-19	4.80

Additionally, as it is indicated in Table 7, the overall satisfaction related to the use of e-learning platforms during COVID-19 equals to 6.33 out of 10 (Q18). A first interpretation of this key figure is that trainees understand and appreciate the value of the alternative mode of the e-learning platform during COVID-19. Thus, their satisfaction seems to be adequate. A logical association would be to investigate if sacrificing the traditional educational approach due to COVID-19 has an impact on this rate. On the other side, the current rate of satisfaction permits us to verify the e-learning mode's positive educational impact.

A supplementary finding of our research is that trainees claim a significant rate for their motivation (6.54 out of 10) to start e-learning during COVID-19 (Q19). Without empirical data to support such claims, we assume that a significant part of their satisfaction is related to their willingness to achieve their educational objectives without interruption from COVID-19 pandemic or even due to their motivation to support their mental health with the continuation of the training.

Furthermore, the average measure for barriers faced during online mode of training delivery equals to 4.80 out of 10 (Q20). This also indicates that the fast transition to online e-learning platforms for medical training, challenged various aspects of the strategy of training. As an average rate, 4.80 indicates that many things could be designed or supported better; on the other hand, it explains the overall satisfaction rate previously discussed.

### Efficiency and Effectiveness of the E-Learning Platform During COVID-19 for Medical Training

In Table 8, we summarize some key aspects related to the efficiency and the effectiveness of the e-learning platform during COVID-19 for medical training. The institutional use of the platform was rated rather low with an absolute value of 50.17 out of 100 (Q15). The overall understanding is that trainees faced difficulties while they were trying to accommodate themselves to the new reality for the delivery of the training due to the COVID-19 pandemic.

**Table 8:** Efficiency and Effectiveness of the E-learning platform during COVID-19 for medical training.

**Question number**	**Rating (1–100)**
Q15. Efficient institutional use of e-learning platform during COVID-19	50.17
Q21. How educative was the use of e-learning platform for you during COVID-19?	57.58
Q22. How effective do you think you can apply the newly learned knowledge, skills or attitude to your daily practice?	59.20

We plan to use this pilot study for designing a new survey targeted to a greater sample in Saudi Arabia for investigating qualitative aspects of this rather fair efficiency. It seems that there is a lot of space for improvements. In the Discussion section, we also use the constructive comments of respondents toward the launch of a theoretical abstraction for the lessons learnt and best practices for effective educational strategies.

The evaluation of trainees for the educative use of the e-learning platform is rather satisfactory: 57.58 out of 100 (Q21). The interpretation of this finding must take into consideration the key qualitative comments of trainees provided in our survey, summarized in the Discussion section.

**Figure 1:**
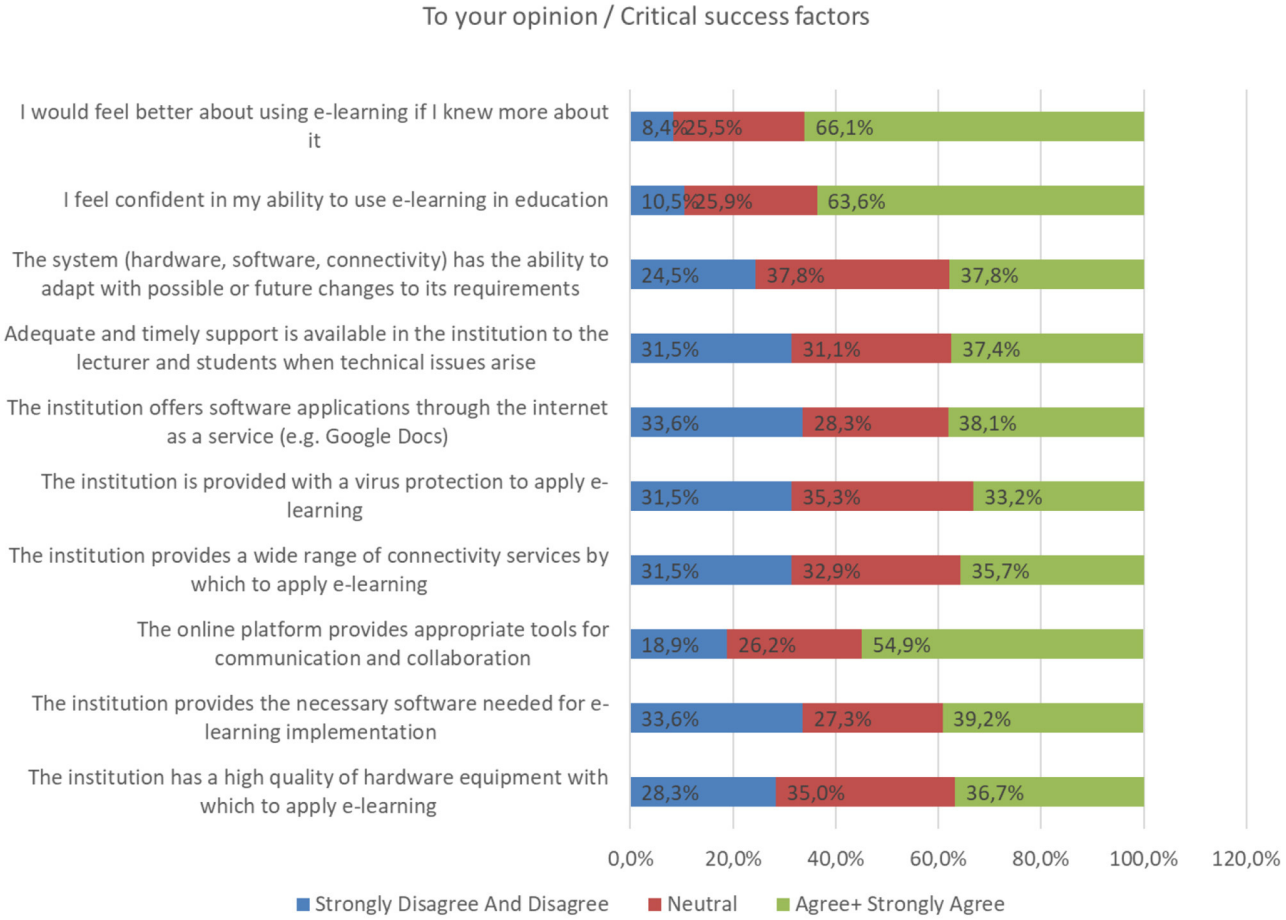
Critical success factors according to the participants' viewpoint.

Next, we will look into particular -mainly technical- parameters, which our trainees deemed as crucial for guaranteeing digital education's productivity. These parameters are assessed by our introducing “to-your-opinion” statements that participants rated with “agree,” “strongly agree,” “disagree,” “strongly disagree,” or “neutral” comments (Table 9; Figure 1).

**Table 9:** Critical success factors according to the participants' viewpoint.

**To your opinion**	**Disagree and strongly disagree (%)**	**Agree and strongly agree (%)**
The institution has a high quality of hardware equipment with which to apply e-learning	28.3	36.7
The institution provides the necessary software needed for e-learning implementation	33.6	39.2
The online platform provides appropriate tools for communication and collaboration	18.9	54.9
The institution provides a wide range of connectivity services by which to apply e-learning	31.5	35.7
The institution is provided with a virus protection to apply e-learning	31.5	33.2
The institution offers software applications through the internet as a service (e.g., Google Docs)	33.6	38.1
Adequate and timely support is available in the institution to the lecturer and students when technical issues arise	31.5	37.4
The system (hardware, software, connectivity) has the ability to adapt with possible or future changes to its requirements	24.5	37.8
I feel confident in my ability to use e-learning in education	10.5	63.6
I would feel better about using e-learning if I knew more about it	8.4	66.1

First, there are some infrastructure issues under the responsibility of medical institutions. The survey's participants were generally dissatisfied or neutral (about two thirds of the sample consisted of “disagree,” “total disagree,” or “neutral” answers) regarding the institutional provision of hardware and software equipment for e-learning use, which includes connectivity services, antivirus programs or internet apps.

We must note that the high rate of neutral answers (~30–35%) is quite concerning and must be further investigated. Most probably, it does not refer to lack of judgement, but on the contrary reflects residents' current incomplete satisfaction, disappointment or even lack of trust in institutional management. In any case, it is fairly evident that “technical support” is considered as a fundamental quality factor for e-learning process and thus, we intend to keep that in mind when planning our recommended model in the discussion section.

Another factor that interests us is the e-learning platform's system fast flexibility, in case of any type of technical problems or future upgrades. About 37% of the residents were fulfilled with the system's aid to such issues. However, there is still plenty of development potential, also given the fact that online education should be advanced enough to handle medical training in long-lasting situations, like the current pandemic. Finally, a little <2 thirds (agree, strongly agree) of trainees are already confident in their competencies required for online lessons, but at the same time would prefer a more thorough education when it comes to skill-building—so that their training is mostly productive.

In the section Discussion that follows, we will also present an integrated interpretation of the lessons learnt and a synthesis of attitudes and opinions communicated by trainees for their medical training COVID-19 experience.

## Discussion

Our study collected significant and valuable data related to the execution of the e-learning method in Saudi Medical Institutions. With the given limitations of our study that are summarized in the section Conclusions, we try to synthesize in a qualitative manner the lessons learnt and the experience gained from the e-learning method in medical training during the COVID-19 pandemic. In this section our focus is on the systematic understanding of the critical success factors of the e-learning method as well as the specification of the core value adding components of the e-learning method. For this purpose the two following subsections provide additional insights. We have to communicate that such an intellectual, qualitative task, results to an outcome that can serve as a basis for further debate rather than as a simple stereotype approach.

### Critical Success Results and Interpretation

After completing a brief interpretation of our research results, we should now target the most essential findings that through discussion will lead us to creating a consistent strategy to strengthen the educational effect of e-learning. So, according to our survey, even though during coronavirus crisis online education's frequency increased, medical institutions were proved to be generally unprepared for fully coordinating the transition process, resulting in:

- Being unable to comply with the academic curriculum and thus, post-poning or skipping over scheduled lectures or training sessions (44%)
- Changes in professional behavior and attitude of both trainees -due to their lack of active interaction- and faculty members, due to their unsuccessful adaptation to e-learning methods (50%)

We also noticed that residents' overall assessment of e-learning experience factors (participation, satisfaction, barriers) was mostly average, however they clearly acknowledged digital education's future potential. From these findings, we deduce that although institutional practice of e-learning was rapid and fulfilled some basic educational standards, the problem lies within regular establishment and training effectiveness of e-learning in the long term.

### Key Pillars of Online Education

In regards to these problematic aspects, in this point we can comment that the basic perceptions of trainees toward the e-learning platforms and the online mode of training can be categorized into nine (9) key dimensions and pillars. These can serve as the basis for a meta-model for Best Practices for E-learning for Residents Training in the COVID-19 pandemic period.

The pillars above are more analytically presented in Table 10 below.

**Table 10:** Key dimensions of online education.

**Technical support**	**Institutional support**
•Improve internet connection (on and off site) •Ensure free subscription to or offers for internet applications•Provide most suitable platforms •Improve technical setup of e-learning •Provide modern tablets	•Ensure free access to medical online facilities for all academic members •Ensure meeting the time schedule •Provide more e-learning tutorials and applications •Store all educational material provided in suitable platforms, for national used (e.g., RCOG) •Provide study rooms (e.g., library) that enable e-learning while at the campus or hospital
**Pedagogy**	**Educational enhancement**
•Strictly comply with academic curriculum and adjust lecture topics •Define clear objectives •Increase motivation •More lectures and activities •More practical sessions •Smart technology enhancement to improve punctuality, interaction with students during lectures and online exams platforms •Divide the sessions into 2 groups: junior and senior	•Build skills and competencies for using e-learning platform •Special workshops, webinars, and training courses for digital literacy •Carefully select/plan mentorships •Promote interaction *via* technological upgrades •Do feedback survey •Pursue Innovation •Encourage international communication
**E-learning platform**	**E-learning strategy**
•Provide daily/weekly sessions •Aim at standardizing online education, even after COVID-19, considering the platforms' complementary impact on medical training •Facilitate registration •Arrange timely subscription to e-learning platforms and offer free use to trainees •Encourage SCFHS interventions for provision of online exams and surgical simulation tools	•Rearrange time schedule: establish minimum break times between lectures and training hours •Do not violate personal (off work) time with post-poned lessons •Encourage e-learning practice if needed for the academic activity •Do not underestimate and underachieve in person teaching approach (Integrate with e-learning) •Promote active engagement in educational process •Accredit e-learning courses or degrees
**Human factors**	**Soft issues**
Ensure residents' effective clinical training in relevance to selected subspecialty •Do not overload trainees with unreasonable medical duties •Ensure that training practice does not intervene with medical education and skill building •Respect trainees' employment rights •Increase social interaction between colleagues •Address work pressure issues •In any case, provide free courses •Additionally, complement in person training with e-learning methods if preferred by trainees	•Instructors: focus on training students with emphasis on motivation, mentorship, acknowledgment, and experiential education •Exhibit professional conduct •Radically address soft issues and adjust academic behavior •Eradicate pathological phenomena in the workplace: discrimination, psychological pressure, abuse, harassment, and any type of misconduct
**Content enhancement**	
•Keep the lectures to-the-point •Focus on group discussion, and interactive learning •Provide medical tutorials •Pose practical questions •Prioritize lecture topics, according to selected specialties •Improve the materials and the applications used for comfortable, efficient and flexible e-learning •Point out more case studies •Purchase more question banks •Seek feedback from trainees •Exploit SCFHS learning material •Hold more webinars •Experiment with new applications •Invest in making the content interesting and archived into free access library	

#### Technical Support

In this category fall various complementary opinions of trainees. Most common concerns related to the availability of internet connection, hardware devices and applications for sophisticated medical activities both in hospital and university grounds. Further technical considerations refer to the availability and cost of different platforms for e-learning and online communication. There is, in general, a demand for offers for both infrastructure and internet service.

#### Pedagogy

One of the most significant pedagogical issues for residents' training during COVID-19 is related to the provision of clear structure of curriculum related topics through e-learning education. This is also associated with the need of residents to have available well-defined and clear training and learning objectives. Additionally, the enhancement of motivations and active engagement of trainees to meaningful learning content is valued as significant. Thus, practical themes related to the availability and planning of more Lectures, pedagogical activities and practical sessions are becoming significant priorities. The same stands for the need of the trainees to be provided with more clear and more structured presentations. From a Smart technology point of view there is a request for enhancements to improve punctuality, interaction with students during lectures and online exams platforms.

#### Institutional Support

The majority of our survey's questionees were either neutral or disappointed (about two thirds in total), when asked about the e-learning support provided by their medical training centers. More specifically, there is a high demand for commonly available facilities, combined with more detailed and flexible time management of the residents' weekly activities. Taking into consideration the physicians' hectic workload, it is quite common that their lecture and working hours may overlap, so under those circumstances they should be excused from their learning sessions or compensate the missed work time on another occasion. Healthcare institutions' e-learning managers should be able to foresee such developments and adjust the existing time schedule. Another issue to be tackled by training centers is the sufficiency and quality of online education software, including modern teaching tutorials, online study guides and antivirus security programs, with the aim of ensuring trainees' professional and efficient training. In parallel to better software provision, there is an additional need for institutions to handle the increasing information load, such as by separating junior from senior's e-learning or establishing platforms in which all educational content can be stored.

#### E-Learning Platform

Careful thought and planning must be put into the operational advancement of the selected e-learning platform. Especially in the COVID-19 era, during which the conference hall is principally substituted by the platform, methodical actions should be taken so as that the learning experience is not degrading. Our study's sample expressed an approximately average satisfaction regarding the use of educational platforms. This fact indicates there is still a lot to be done when it comes to the functionality of the e-learning platform. Above all, we need internet connection stability which can be assessed with numerous webtools and is a must for establishing an uninhibited and time-saving educational process. Residents should, also, be able to attend their training sessions on a daily basis by enrolling easily on the e-learning platform in an automatized manner, if possible. A very promising prospect for the extension of the potential capacities of the e-learning platform would be the establishment of online examination or even medical operations' simulation -using the existing platform, altogether arranged by Saudi Commission for Health Specialties. Last, even when not highly needed, e-learning platforms can always be used in combination with traditional teaching methods.

#### E-Learning Strategy

Next, even if all the essential requirements pertaining to the technical use of e-learning software are met, we must also apply some basic principles of how and under which limitations to use the e-learning platform, thus we ought to structure an accurate and specified “e-learning strategy.” In order to clarify and explain further the composition of such a strategy, we will mention a few examples of which components we deem important. First, the teaching process must always take place only during academic-engaged time, that is the time spent at hospital or university grounds or by extension the time aimed strictly at learning; in other words there should be no intentional involvement in training physicians' private time at home. Second, it is necessary that we ensure there is a minimum set break time period between working hours and lecture hours, which will be used for physical and digital transportation to the e-learning environment, as well as for the residents to have a decent rest. By the same logic, various more standards -including academic recognition of e-learning and importance of physical education- can be established, always to guarantee educational efficacy.

#### Educational Enhancement

Considering that teaching is a form of bidirectional communication, we aim to preserve this particular characteristic when “passing through the screen” in online education. More specifically, as shown in this study, residents would like to and basically need to know more about what they can do and how they can interact *via* e-learning. These requests pose the challenge of interactive e-learning and skill-building activities, which are essential for simulating physical education. Toward this direction, special workshops, webinars, and tutorials can be realized, educating physicians in terms of technical skills, e-learning literacy, mentorship and communication. The contribution of state-of-the-art technology to educational enhancement is also of the essence.

#### Human Factors

In order to optimize the e-learning experience, our research also focuses on dealing with particular human factors, which are rather subjective and vary among trainees and healthcare institutions, however they offer a general concept of the trainees' needs and how we must proceed to sufficiently satisfy them. Human factors mostly incorporate great demand of direct social interaction and free online courses within the academic duty hours, as well as fairer distribution of workload and time schedule. There has also been notice of institutional deficiency, in relation to providing residents with practical medical knowledge, which is independently obtained most of the times, as reported. Such issues must be tackled, by systematically and directly addressing the trainees' anxieties and problems, e.g., using e-learning platform's questionnaires.

#### Content Enhancement

The goal of online education, as it currently works in the COVID-19 pandemic, is to temporarily substitute -in the most efficient way possible- in-person education, due to an emergency situation. Nevertheless, future prospects should to at increase e-learning quality and viability by fundamentally rearranging and enriching educational content. Main themes of content enhancement are the improvement of teaching material and applications, coupled with running more innovative lessons, based on group discussion, active participation, instructive medical tutorials, and above all free access to educational material. The topics of the lectures should also be more precise, to-the-point and relevant to trainees' needs -particularly to their selected specialty and up-to-date medical data (articles, webinars etc.)- so that the lesson has a practical use and the knowledge provided is well-rounded.

#### Soft Issues

Similar to human factors, soft issues include experiential concerns of highly subjective nature, that residents express about the academic behavior of their medical trainers or instructors during both lectures and training sessions. Problematic situations, in which trainers exhibit professional deficiency, negligence, and indifference toward their duties or even overcharge their subordinates in terms of routine medicals tasks, should be addressed. Online education must also be rid of more severe issues, such as work intimidation and harassment of any type.

### Our Contribution: E-Learning Quality Enhancement Model and Maturity Model

**Figure 2:**
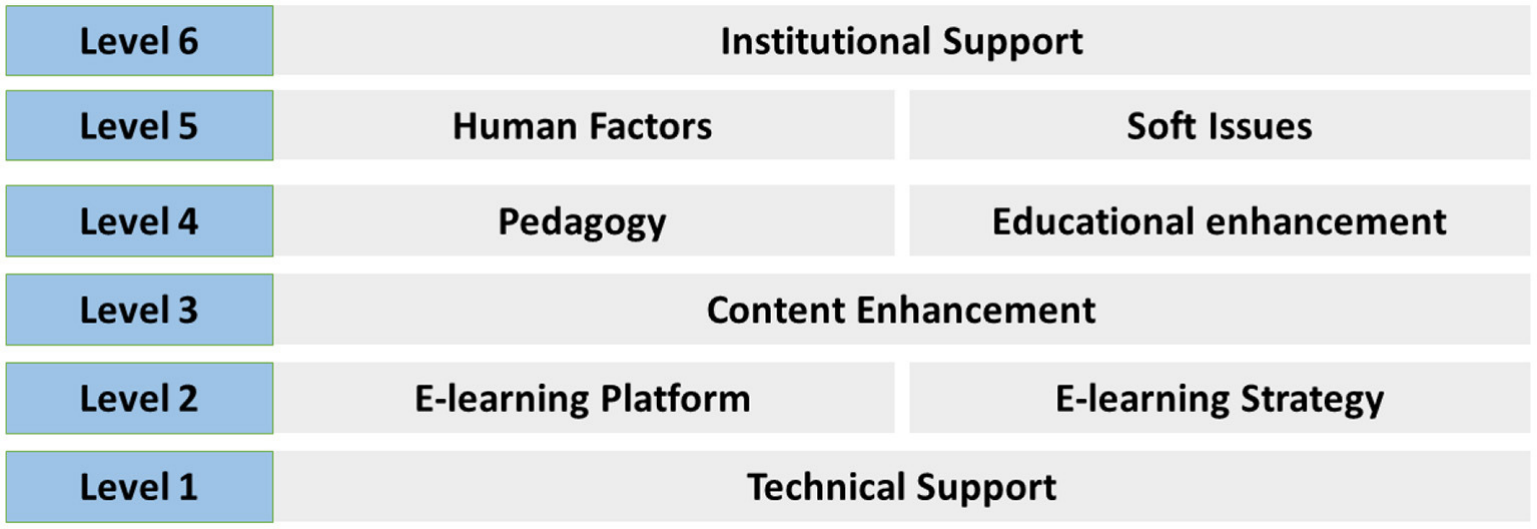
E-learning Quality enhancement model.

Based on the analytical categorization of the nine key pillars, mentioned above, in this section we will proceed with introducing a new, multi-functional, strategic model for “Quality enhancement of e-learning process in medical institutions during COVID-19 pandemic.” This model, displayed at Figure 2, consists of six priority levels, the necessity of each decreases from bottom to top. Level 1 contains technical support; level 2 includes e-learning platform along with e-learning strategy; level 3 contains content enhancement and so on. For levels that include more than one parameter, each one has equal value to the other.

The above model is also one of our research team's main contributions in the context of restructuring online education during COVID-19 pandemic and is realized by analyzing our research goal into key dimensions and then classifying those into defined levels. As for its value, the recommended strategy can be utilized in various ways. First of all, it can be implemented by medical institutions and medical training centers as an operational framework for improving the existing e-learning methods and maximizing the effectiveness not only of typical teaching with lectures but also of healthcare simulation courses.

Toward this purpose, the model can, also, serve as a foundation for complementary restructuring actions, aimed at preserving the accomplished enhancement of the e-learning process. It would be very beneficial that medical directors or instructors in charge of e-learning arrange a regular assessment of the model's key parameters through open dialogue or indirect interaction with the trainees—using, for example, the existing polling tools of e-learning platforms or even questionnaires that maintain residents' anonymity. As a result, any complaints or unfulfilled goals will be reported and if possible addressed, so that there is a tactical review of the model.

Another critical effort would be the establishment of online education as a mandatory tool not only during the COVID-19 era but also in the long-term, as our findings also confirmed. Undoubtedly, in-person education is irreplaceable regarding its educational value, still e-learning can be: first, necessary in emergency situations such as the current pandemic; and second, plenty helpful as a supplemental teaching method under normal circumstances.

However, redeveloping online learning structure and strategy cannot possibly be an immediate change, because of a lack of infrastructure, resources, human force, and probably time availability. Still, we acknowledge the frequent necessity of instant e-learning use, as it is at present due to the coronavirus crisis. Thus, we need an additional theoretical model, which accompanies the one introduced in Figure 2. To be more specific, in the previous model we examined e-learning enhancement, according to defined levels (one to six), which in fact are comprised of one or two specific key pillars (technical support, institutional support, etc.). At this point, we need to look into the development of e-learning, setting priority measures in each “level,” thus defining “stages.”

**Figure 3:**
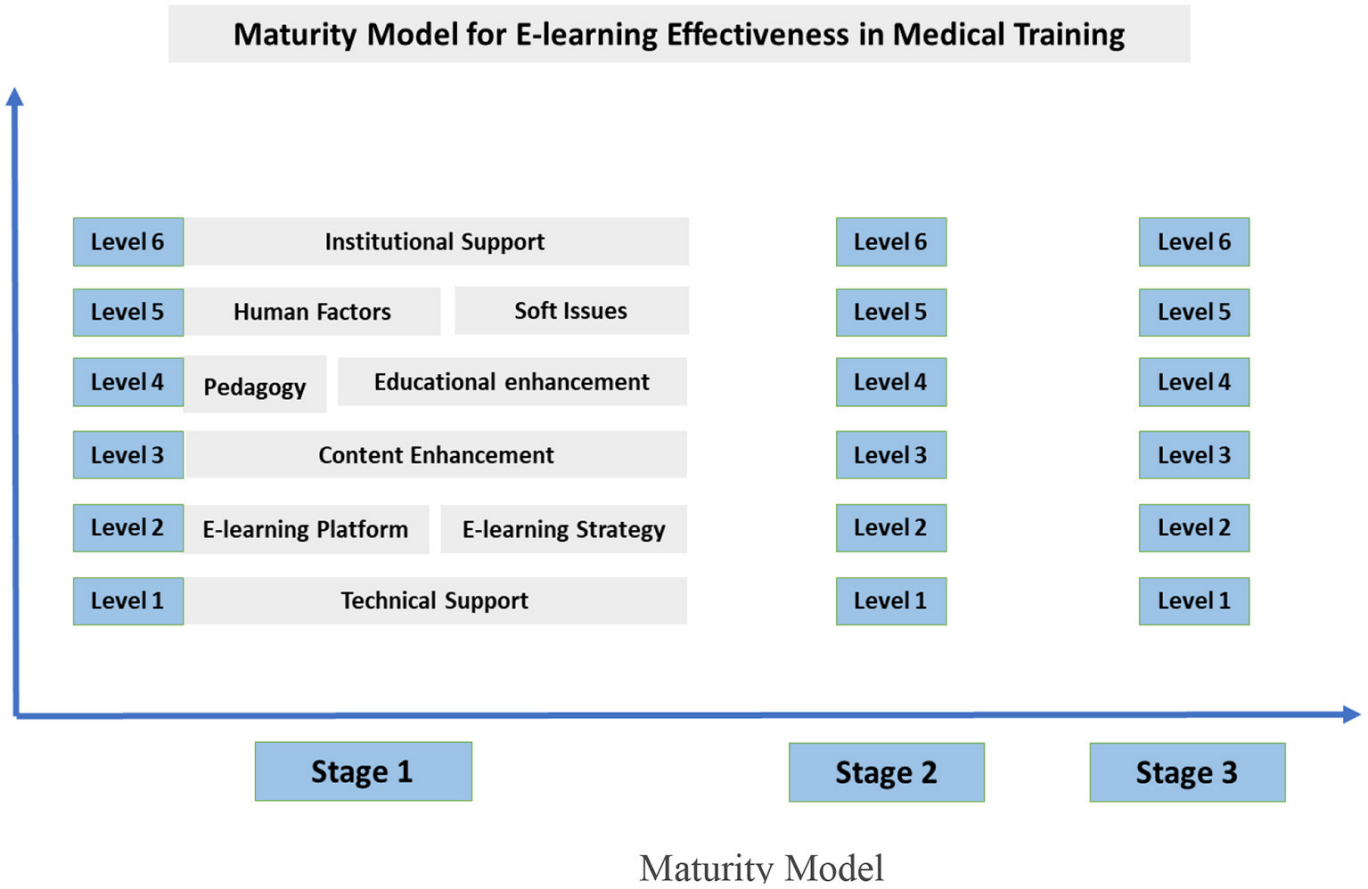
Maturity Model.

In other words, the improvement of online education in medical institutions can be studied as a temporally-evolving developmental process and be divided into “stages.” Since there is a need for fast development, any upgrading efforts should start with “stage 1” for example -so that there is a prompt result- and, if finished, move on to the next, less crucial stages. Based on this reasoning, we suggest a “Maturity Model for E-learning Effectiveness in Medical training” (Figure 3), capable of optimizing e-learning impact and value and offering practical guidelines on how to proceed to each stage.

In Figure 3, we can see our Maturity Model's basic components:

1. We define three (3) “Maturity stages,” all of which require fulfilling specific measures of every key pillar (levels one to six).
2. When the model is applied, priority is given to the earlier stage and lowest level, that is stage 1, level 1. Moving on to the next stages, theoretical effectiveness of online education for medical trainees increases.

**Figure 4:**
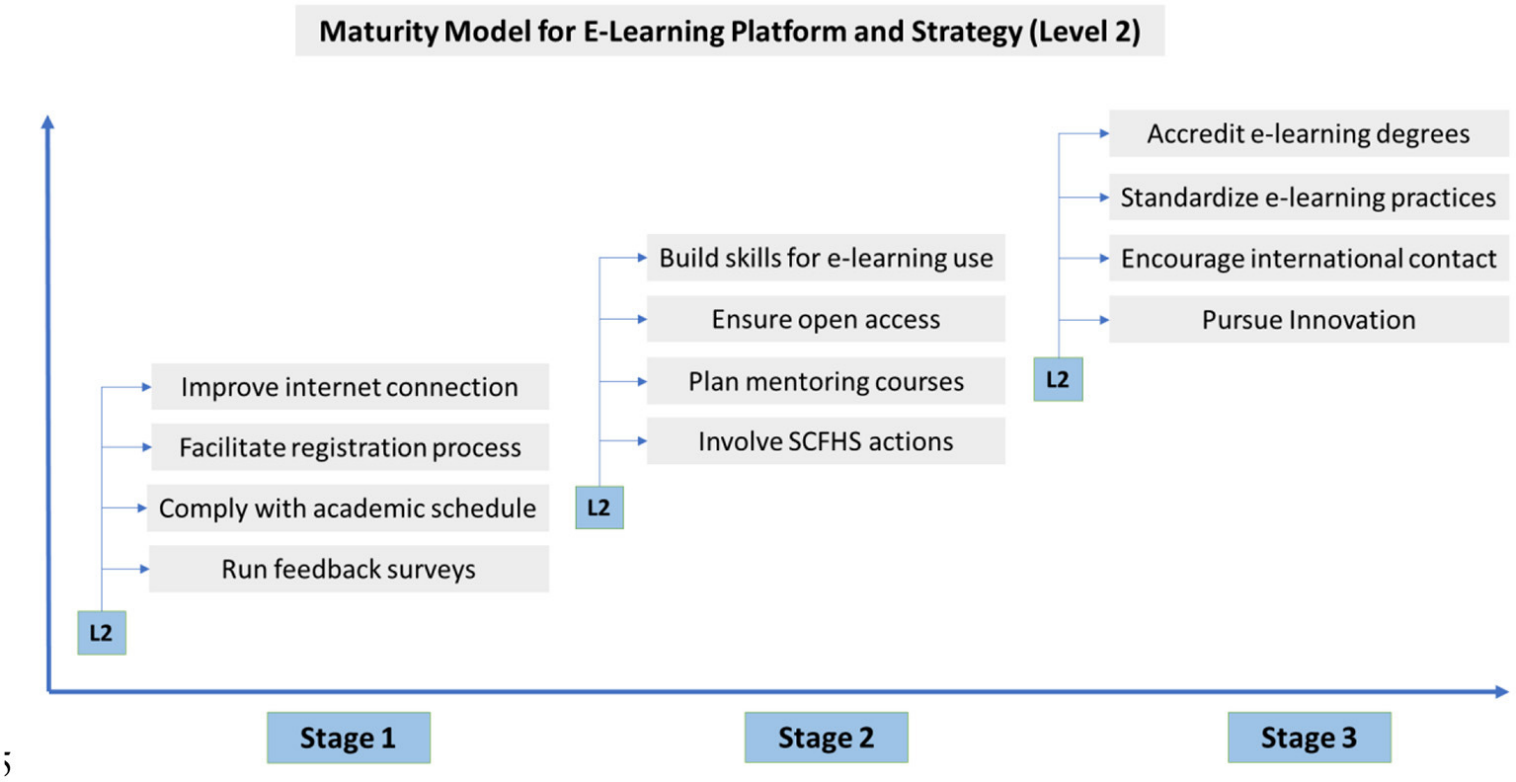
Maturity Model for Level 2.

**Figure 5:**
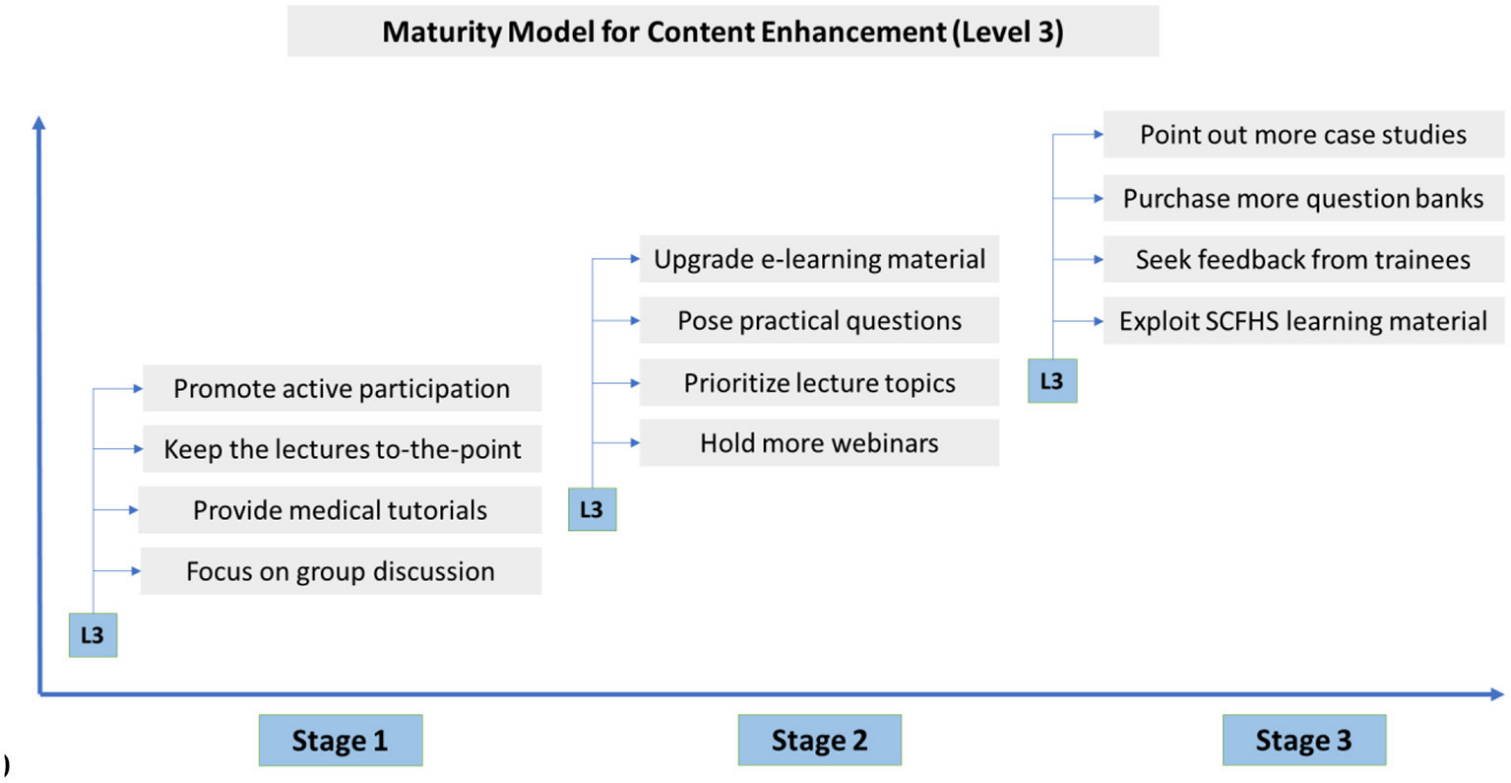
Maturity Model for Level 3.

In order to demonstrate our strategy's concept in a more comprehensive way, we provide two indicative models (Figures 4, 5) which refer to specific measures for the development of e-learning platform and strategy (L2) and content enhancement (L3) accordingly.

To sum up, we firmly believe that combining Quality enhancement model with Maturity Model can serve as a management tool and therefore, turn residents' experience of online education into optimal medical practices.

## Conclusion

In conclusion, this research investigated the effectiveness of the e-learning approach, used by numerous medical institutions in Saudi Arabia, in relation to all types of emerging obstacles in the medical training and teaching process: limited institutional readiness, technical difficulties, behavioral and psychological concerns, soft issues, lack of e-learning content and tools, and—above all—academic efficacy. Our resolution strategy was based on accurate definition of e-learning factors—according to residents' viewpoints, and further analyses on nine key dimensions. After processing the results of the analyses, we constructed two practical models, which associated online education's effectiveness first, with priority levels (Quality enhancement Model), and secondly, with time stages (Maturity Model). the main purpose of both is the time and cost-effective implementation of e-learning in medical institutions of higher education.

However, we clearly acknowledge this study's limitations. First of all, it is evident that any conclusions made were based on the sample of 300 survey participants. Thus, results regarding e-learning support, engagement, and overall satisfaction are subject to modulations among training centers and even individual trainees. Furthermore, we recognize that the suggested models have not directly considered financial parameters that restrict transition from one step to another. Still, we provide practical guidance on e-learning priorities, that may facilitate institutions' cost management. Other limitations of this study are the issues of online data protection against piracy or illegal recording of provided lectures, most of which should be addressed in cooperation with governmental legislature.

Despite the limitations, the strategy posed by this research could be a foundation for converting online education into a more consistent, independent, and long-lasting learning approach, capable of fully supporting in-person education, if needed. This would also be one of our main future concerns, along with expanding our research to a larger population.

Additionally, in the future directions for research we are interested in linking our theoretical models to a machine learning approach that will allow adjusting customization of learning content in e-learning platforms based on learners' profiles. Also, the integration of sophisticated artificial algorithms for investigating and recommending learning paths to learners based on their preferences. Last but not least, group-assessment strategies and investigation of key success factors for group-based active e-learning is another key direction for future research.

As an overall conclusion, our research contributes two models to the body of knowledge of e-learning for medical education, which associate e-learning training effectiveness firstly, with core-capabilities/priority levels (Quality enhancement Model) and secondly, with time stages (Maturity Model). We do believe that these contributions can be used as a scientific contribution capable of initiating diverse dialogue from communities of computer engineers, e-learning specialists, medical experts, and content providers.

## Data Availability Statement

The raw data supporting the conclusions of this article will be made available by the authors, without undue reservation.

## Ethics Statement

The studies involving human participants were reviewed and approved by Saudi Commission for the Health Specialty Ethics Committee. The patients/participants provided their written informed consent to participate in this study.

## Author Contributions

All authors listed have made a substantial, direct and intellectual contribution to the work, and approved it for publication.

## Conflict of Interest

The authors declare that the research was conducted in the absence of any commercial or financial relationships that could be construed as a potential conflict of interest.

## Publisher's Note

All claims expressed in this article are solely those of the authors and do not necessarily represent those of their affiliated organizations, or those of the publisher, the editors and the reviewers. Any product that may be evaluated in this article, or claim that may be made by its manufacturer, is not guaranteed or endorsed by the publisher.
